# Optic disc parameters and associations with early life exposures in over 3000 12-year-old children: findings from the ALSPAC cohort

**DOI:** 10.1038/s41433-025-03716-2

**Published:** 2025-02-22

**Authors:** Alexandra L. Creavin, Kate Tilling, Nicholas Timpson, Cathy EM Williams

**Affiliations:** 1 Clinical Lecturer, Bristol Medical School, University of Bristol, Bristol, UK; 2 Professor of Medical Statistics, Bristol Medical School, University of Bristol, Bristol, UK; 3 Professor of Genetic Epidemiology, Bristol Medical School, University of Bristol, Bristol, UK; 4 Professor of Paediatric Ophthalmology, Bristol Medical School, University of Bristol, Bristol, UK

**Keywords:** Risk factors, Predictive markers

## Abstract

**Objectives:**

We aimed to investigate the distribution of small optic discs and large cup-to-disc ratio in children and to examine associations with maternal and environmental factors.

**Methods:**

Retinal photographs were graded from over 3000 12-year-olds in the Avon Longitudinal Study of Parents and Children. Regression models examined associations between disc parameters and maternal and early-life exposures.

**Results:**

Mean cup-to-disc area ratio (CDAR) for 3288 children was 0.21 (95%CI 0.20,0.21). Discs with CDAR > 0.3 were present in 11%. The odds of CDAR > 0.3 were increased nearly three-fold in underweight children (adjusted odds ratio (aOR) 2.9 (1.1, 7.3) *p *= 0.03) and 28-fold in severely premature ( < 28 weeks) children (paOR 28 95%CI 4.6,172, *p *< 0.001) with nearly one in four children affected. Mean cup-to-fovea/disc diameter (CF/DD) for 3327 children was 2.48 (95%CI 2.47,2.50). Small discs (CF/DD > 3) were present in 6% of which a third were bilateral. The odds of a small disc were increased in the offspring of mothers who smoked in pregnancy (aOR 1.7 (1.0,2.8) *p *= 0.04) and more than doubled in children born with a small head circumference (aOR 2.5 (1.4,4.5) *p *< 0.001).

**Conclusions:**

Small optic discs and high cup-to-disc ratio are more frequent than usually supposed at age 12. The odds of CDAR > 0.3 are increased by severe prematurity and pathologically low child BMI. The odds of a small disc are increased by maternal smoking and small head circumference. Optimisation of risk factors in pregnancy and delivery and early childhood nutrition may play an important role in ophthalmic neurodevelopment and thus have a lifelong impact on ocular health.

## Introduction

In adults, an increasing cup size relative to the optic disc signifies nerve fibre loss, aiding disease diagnosis and monitoring, with a cup-to-disc ratio (CDR) > 0.3 indicating potential glaucoma [1]. However, defining normal versus abnormal thresholds in children, and interpreting asymmetry, remains uncertain. No evidence-based guidelines exist for managing children with large CDR, often leading to referrals to hospital services following routine optometric examination.

**Fig. 1:**
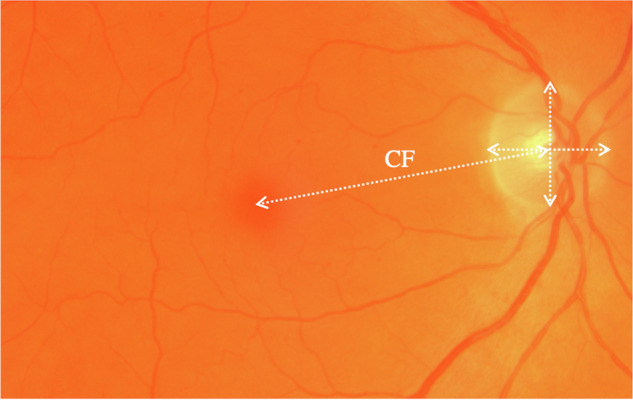
Retinal Image illustrating CF/DD. Ratio of the distance from the centre of the disc to the fovea (CF), divided by disc diameter: CF/DD.

The cup-to-fovea over disc diameter (CF/DD) is a dimensionless measure that can be used in identifying a small disc: suspected when CF/DD is greater than 3.0 and very likely if greater than 4.0 [2–4]. This measure has been used to identify isolated small optic discs and eyes at risk of optic nerve hypoplasia from retinal photographs [3]. Figure 1 illustrates measurement of CF/DD. In cases of optic nerve hypoplasia visual acuity may range from no perception of light to normal [5].

Quantification of optic nerve head damage occurs in the context of many other clinical measures such as visual field tests and intraocular pressure. However, these can prove challenging in children, as measurement of visual fields shows poor reliability in those younger than eight, and nerve visualisation and intra-ocular pressure measurement can be hampered by patient movement and lid squeezing, particularly when children are experiencing other neurodevelopmental problems [6]. While optical coherence tomography (OCT) aids diagnosis and monitoring, paediatric versions aren’t universally accessible, sustaining the use of cup-to-disc ratio (CDR) and retinal photography globally. In practice, children with high cup-to-disc ratio or small optic discs often present a clinical dilemma [7]. Enhanced understanding of optic disc parameters in infants and children is crucial for diagnosing and monitoring ophthalmic and neurological diseases, minimising unnecessary referrals and investigations, while emphasising early detection to optimise visual, academic, and social outcomes.

This study employs fundus photography-derived optic disc data from a substantial cohort to explore optic disc parameter distributions (CDAR and CF/DD) in children, gauging the prevalence of paediatric high cup-to-disc ratio or small discs and their associations with maternal and environmental factors.

## Subjects and methods

We conducted an analysis of digital retinal photography data from the Avon Longitudinal Study of Parents and Children (ALSPAC), a population-based cohort study [8, 9]. Pregnant women residing in Avon, UK, with expected delivery dates between April 1, 1991, and December 31, 1992, were invited to participate. Initially, 14,541 pregnancies were enrolled, resulting in 13,988 children alive at one year of age. For analyses after the age of seven, the total sample size was 15,447 pregnancies, with 14,901 children alive at one year of age [8, 9]. Data on early-life and maternal exposures were collected through self-completion questionnaires, obstetric medical notes, observation, clinical examination, and biological samples. The study website contains details of the data through a searchable data dictionary and variable search tool [10]. Not all participants with retinal photographs completed all ALSPAC clinics and questionnaires and so varying numbers of participants had data for each exposure.

Retinal photographs were taken at 12 years of age using a non-mydriatic Topcon camera. Attendees for retinal imaging were compared to non-attendees. Optic nerve images were graded monoscopically using semi-automated software developed by Professor James Morgan (StereoDx, Cardiff University) [11]. The trained graders were blinded to clinical information. The cup-to-disc area ratio (CDAR) and the cup-to-fovea over disc diameter (CF/DD) were automatically generated after manual placement of dots around the cup perimeter and on the fovea, respectively (see Fig. 1). Both colour and red-free photographs were taken to facilitate identification of the optic disc boundaries and foveal position. The foveal centre was identified by the grader using the light reflex to help judge position. The average disc diameter was calculated by adding the vertical and transverse disc diameters and then dividing by 2. Half the average diameter of the optic disc was added to this distance to obtain the ‘disc-macula distance’.

Seven technicians graded the images, requiring consideration of systematic differences in measurements due to grader identity. After training, an agreement exercise was performed on a subset of unique and repeat images representing a range of grading ease and disc parameters. Grader 3 did not complete the agreement exercise and so could not be included. The two-way random effects, absolute agreement, multiple intraclass correlation coefficient (ICC, (2, *k*)) with 95% confidence intervals was used to determine the variability between graders when grading the same image.

Bland-Altman plots and analyses of variance (ANOVA) were used to compare graders to a reference standard (the lead optometrist for the glaucoma-shared care service). Tukey’s procedure was applied to pairwise comparisons. The label “Grader 8” was used where grader identify could not be ascertained (*n *= 31).

To mitigate confounding and reduce the standard error of estimates, covariates hypothesized to be associated with either CDAR or CF/DD and small discs in the context of optic nerve hypoplasia were identified a priori from literature and expert discussions. Table 1 gives details of the measurement of outcome, exposure, and confounder variables.

**Table 1:** Variable categories and measurement.

Variable	Method of measurement	Categories
*Optic disc measures*		
Area CDR	Semi-automated measurement by grader according to protocol	Normal ≤ 0.3 | Abnormal >0.3
CF/DD	Semi-automated measurement by grader according to protocol	Normal ≤ 3 | Abnormal >3
*Maternal factors*		
Primiparity	Maternal report via questionnaire at 18 weeks and review of medical notes	Primiparous | Multiparous
Education	Maternal report via questionnaire at 32 weeks’ gestation	Completion of CSE/O levels | Completion of A levels or degree
Age at delivery (years)^a^	Obstetric clinical records	<20 years | 20–34.9 | 35–40 | >40 years
Smoking in first trimester	Maternal report via questionnaire	No | Yes
Alcohol in first trimester	Maternal report via questionnaire at 18 weeks	None | <1 per week | <1–2 | >2 per week
Weight loss in first trimester	Measurement of weight extracted from obstetric medical notes	None | Weight lost
Body mass index	Measurement of baseline height and weight extracted from obstetric medical notes	<18.5 | 18.5–24.9 | 25–29.9 | >30
*Child factors*		
Prematurity (weeks)	Gestational age recorded at birth in obstetric clinical records and reported in maternal questionnaire	37+ | 33–36 | 28–32 | <28 weeks
Birth weight (grams)	Obstetric clinical records and maternal questionnaire	≥ 2500 | <2500 grams
Birth head circumference (cm)^b^	Obstetric clinical records and maternal questionnaire	Girls: <33.5 | 33.5–36 | >36 || Boys: <33.4 | 33.4–36.5 | >36.5
Birth length (cm)	Obstetric clinical records and maternal questionnaire	Girls: <49 | 49–56 | >56 || Boys: <49.6 | 49.6–55 | >55
Child BMI	Measurement of height and weight in clinic at age 11 years	Girls: <14.4 | 14.4–21.75 | >21.75 || Boys: <14.5 | 14.5–21.0 | >21.0
Child ethnicity	Maternal report in questionnaire	White | Other
Visual acuity	In clinic at 11 years. Monocular LogMAR acuity in habitual state and with pinhole	Worse than 0.3 in both eyes, one eye, or neither eye

^a^<20 years was compared with 20–40 years when young maternal age was the exposure.

^b^Small head circumference compared with normal head circumference when small head circumference was the exposure.

Data cleaning and analyses were conducted using Stata version 13. The second delivered twin was excluded from analyses in the case of multiple births. Outcome data were analysed as ratios based on unit differences. Left and right eyes were compared, and right eyes were used in subsequent analyses. The distribution of optic disc parameters was described using means, medians, and ranges. Maternal and child-related factors hypothesized to be associated with CDAR or with a small optic nerve head were explored using stratification, chi-squared tests, and regression models incorporating inverse probability weighting to mitigate bias from missing data. The weighting model used logistic regression to relate case completeness to baseline variables: maternal age, gestation, sex, maternal smoking, and parity and was used to estimate the probability of being a complete case for each person.

### Power calculation

A subset of 1250 ALSPAC photographs had previously been used to calculate a mean CDAR of 0.3 (SD 0.08). Analysis of approximately 3300 images had 80% power with a significance of 0.05 to detect a minimum difference in means of 0.01 in CDAR for exposures affecting 15% of participants (e.g., maternal smoking) and a difference in means of 0.014 for exposures affecting 5% of ALSPAC participants (e.g., preterm birth).

## Results

### Attendees versus non-attendees

**Fig. 2:**
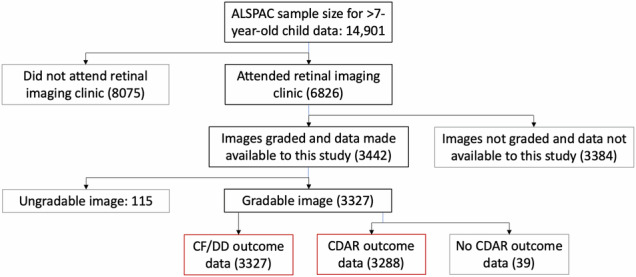
ALSPAC participants.

Figure 2 is a flow diagram of participants. We compared individuals attending retinal photography and achieving gradable images with those who did not. Children with photographs were more likely to be white, female, term-born, with normal birth weight, and have highly educated, non-smoking, older mothers (see Supplementary Table 1: Socio-demographic characteristics, birth outcomes, and visual outcomes of participants attending and included in the analysis versus non-attending children). Both groups exhibited rare poor visual acuity and maternal heavy drinking.

### Sample description

A gradable retinal image was obtained from 3327 individuals, averaging 12.9 years old (SD 0.27, range 11–14 years). Females constituted just over half (50.7%), with 96.0% reporting white ethnicity, 95.5% born at term, and 95.8% with normal birth weight. Approximately 17.3% were born to mothers who smoked in the first trimester. Instances of heavy drinking during pregnancy (0.7%) and mothers over 40 years old (1.5%) were rare.

### Rater agreement

Inter-grader agreement was good for CDAR (ICC (95%CI) 0.74 (0.62, 0.84)) and moderate for CF/DD (ICC (95%CI) 0.57 (0.42, 0.72)). Repeatability was good for both CDAR (ICC (95%CI) 0.77 (0.59, 0.89)) and CF/DD (ICC (95% CI) 0.78 (0.61, 0.89)). Tukey post hoc test and ANOVA revealed slight underestimation of optic disc parameters by graders, particularly for larger measurements, compared to the reference standard.

### Distribution of optic disc parameters

#### Cup-to-disc area ratio

CDAR measures were collected from 3288 children. Among those with gradable images in both eyes (*n *= 3078) CDAR was similar in 99.5%: 92% showed ≤0.1 difference between eyes, 8% >0.1 but <0.2, and 0.5% ≥0.2.

**Fig. 3:**
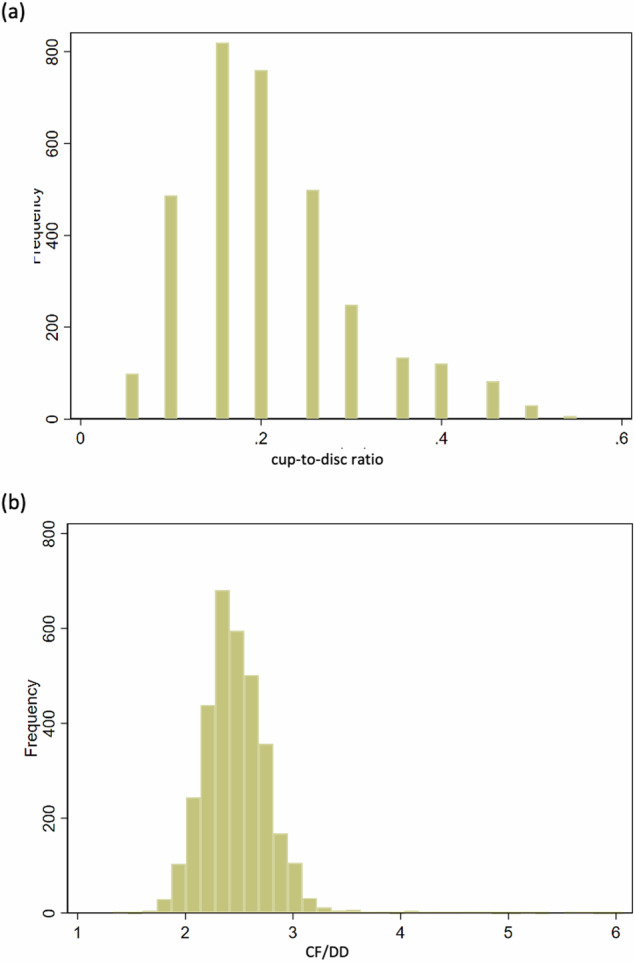
Histogram of distribution of optic disc parameters in the study participants. (**a**) CDAR (**b**) CF/DD Histogram of (**a**) CDAR and (**b**) CF/DD distribution in the ALSPAC.

Mean (standard deviation (SD)) CDAR was 0.21 (0.09), median 0.20 and range 0.05 to 0.55. Mode CDAR was 0.15 (*n *= 842). CDAR distribution was right skewed (see Fig. 3). Male and female mean (sd) CDARs were comparable: 0.21 (0.1) and 0.20 (0.1) respectively. Large CDAR ( > 0.3) prevalence was 11% (*n *= 367).

#### Cup-to-fovea over disc diameter

CF/DD measurements were obtained from 3327 children. Mean (SD) CF/DD was 2.48 (0.36), median 2.45 and range 1.33–6.06. Mode CF/DD was 2.49 (*n *= 65). CF/DD followed a normal distribution (see Fig. 3). Male and female CF/DD were comparable: 2.48 (0.4) and 2.47 (0.4) respectively. Small optic disc (CF/DD > 3) prevalence was 6% (*n *= 212) with one third bilateral (*n *= 75).

### Relationship with exposures

Table 2 presents uni- and multi-variable logistic regression models for (a) CDAR > 0.3 and (b) CF/DD > 3 against each predetermined exposure.

**Table 2:** Univariable and multivariable logistic regressions for (a) large CDAR and (b) large CF/DD (small OD) against predetermined exposures.

	N^a^	Category	Unadjusted			Partially adjusted & weighted^b^			Fully adjusted & weighted		
			OR	95% CI	P	OR	95% CI	P	OR	95% CI	P
(a) CDAR											
Birth weight^c^	2423	Low	1.04	0.60, 1.80	0.89	2.09	0.65, 6.71	0.22	0.67	0.11, 4.08	0.67
Maternal smoking^d^	2612	Smoker	0.81	0.59, 1.11	0.18	0.86	0.48, 1.55	0.62	0.93	0.49, 1.76	0.83
Very preterm^e^	2652	Very premature	2.80	1.08, 7.04	0.03	28	4.62, 172	<0.001	-	-	-
Head circumference^f^	2030	Small	0.94	0.65, 1.35	0.75	0.76	0.44, 1.32	0.33	0.67	0.36, 1.24	0.20
Head circumference^g^	2391	Large	1.30	1.02, 1.65	0.03	1.59	1.02, 2.49	0.04	1.32	0.84, 2.07	0.23
Low child BMI^h^	2622	Low	0.85	0.42, 1.71	0.65	2.14	0.91, 5.01	0.08	2.87	1.13, 7.27	0.03

(b) CF/DD											
Birth weight^c^	2424	Low	2.49	1.15, 4.17	0.02	1.35	0.58, 3.14	0.48	1.35	0.34, 5.40	0.67
Head circumference^f^	2652	Small	1.82	1.14, 2.90	0.01	2.25	1.31, 3.86	<0.001	2.49	1.37, 4.51	<0.001
Head circumference^g^	2652	Large	1.56	1.09, 2.25	0.02	1.25	0.77, 2.01	0.37	1.18	0.70, 1.99	0.53
Maternal alcohol^i^	2855	Regular/heavy	0.77	0.46, 1.29	0.32	0.65	0.36, 1.16	0.15	0.66	0.37, 1.18	0.16
Maternal smoking^d^	2617	Smoker	1.44	0.95, 2.20	0.09	1.55	0.96, 2.50	0.07	1.69	1.01, 2.84	0.04
Very preterm^e^	2652	Very premature	2.20	0.51, 9.50	0.29	-	-	-	-	-	-
T1 weight loss^j^	2652	Loss	1.20	0.48, 3.00	0.70	0.61	0.19, 1.95	0.41	0.66	0.20, 2.10	0.48
Maternal age^k^	2526	Young	2.08	0.82, 5.31	0.12	0.90	0.19, 4.29	0.89	0.90	0.19, 4.29	0.89
Parity^l^	2868	Multiparous	0.86	0.61, 1.23	0.42	1.09	0.74, 1.60	0.68	1.12	0.75, 1.67	0.58

^a^N in fully adjusted model; Partially adjusted for sex, grader, ethnicity.

^b^Odds ratio (OR), 95% confidence intervals (95CI). Dashes indicate when numbers in each group were insufficient to run the model.

Fully adjusted model was adjusted for (M = maternal, B = birth):

^c^M alcohol, M smoking, M BMI, M weight loss in 1^st^ trimester, B head circumference, B length, preterm B.

^d^M alcohol, M parity, M age, M BMI, M education.

^e^M alcohol, M weight loss in 1^st^ trimester, M smoking.

^f^Preterm B, M alcohol, M smoking, M weight loss in 1^st^ trimester.

^g^Preterm B, M alcohol, M smoking, M weight loss in 1^st^ trimester.

^h^M BMI, M education.

^i^M education, M age.

^j^M alcohol.

^k^Nil.

^l^M education, M age.

#### CDAR

The odds of high cup-to-disc ratio were comparable across groups for birth weight, maternal smoking, and head circumference at birth. Severely premature children born before 32 weeks’ gestation had 28 times higher odds of large CDAR at age twelve (partially adjusted odds ratio (paOR) (95% confidence interval (CI)) 28 (4.62, 172) *p *= <0.001). Numbers were insufficient to run the fully adjusted model. Children with a low BMI at age eleven had three times higher odds of large CDAR (adjusted odds ratio (aOR) 2.87 (1.13, 7.27) *p *= 0.03).

A high cup-to-disc ratio was identified in over a quarter (26%) of children born before 32 weeks (*n *= 6) compared to just 8% of moderately preterm (*n *= 9) and 11% of term children (*n *= 339) with evidence of a difference between groups (*p *= 0.012).

#### CF/DD

The odds of a small disc were comparable across groups for birth weight, large head circumference, maternal alcohol, preterm birth, weight loss in trimester one, maternal age, and parity. Children with a small head circumference at birth had more than twice the odds of a small optic disc at age twelve (aOR 2.49 (1.37, 4.51) *p *= <0.001). A small optic disc was found in 7% (*n *= 25) of those born with a small head circumference, 6% (*n *= 52) with a large circumference and 4% (*n *= 75) with a normal circumference (*p *= 0.009).

Children born to mothers who smoked in pregnancy also had increased odds of a small disc (aOR 1.69 (1.01, 2.84, *p *= 0.04). There was a lack of association with some exposures hypothesized to cause a small optic disc in the context of optic nerve hypoplasia: first trimester weight loss (aOR 0.66 (0.20,2.10)), maternal age (aOR 0.9 (0.19,4.29) *p *= 0.89) and parity (aOR 1.12 (0.75,1.67) *p *= 0.58).

## Discussion

### Strengths and limitations

The ALSPAC study provides rich life course data for many participants across a geographic region, offering sufficient power to investigate rare exposures, though some adjusted analyses may have limited power. However, the lack of representation from ethnic minorities and less affluent families may restrict the generalisability of findings [8].

Self-reported data such as smoking or alcohol intake are susceptible to misreporting, potentially biasing associations towards null values. Observer variability and measurement errors may affect physical measures, despite training and protocols.

Retinal photography, while widely used in clinical practice, faces challenges in optic disc measurement due to landmark identification and magnification error, mitigated by using dimensionless ratios like CDAR. Retinal photography and OCT have been demonstrated to yield comparable results with relative measures, though absolute measures may be around 10% smaller using photography [12].

Monoscopic software usage may underestimate CDAR compared to stereoscopic methods, reducing prevalence estimations for enlarged cup-to-disc ratios [11]. Although graders tended to underestimate parameters, their reasonable agreement questions the necessity of a reference standard with inherent errors. A priori analysis protocols aimed to minimize bias. Residual confounding from unmeasured exposures remains possible.

### Interpretation

The average 12-year-old child typically exhibits a CDAR of around 0.2, regardless of gender, similar to findings in Australian six-year-olds [13]. While a cup-to-disc ratio >0.2 has been reported to occur in fewer than 1% of children our study shows a prevalence of CDAR > 0.3 in 11% of participants, suggesting a potential need to reassess the clinical implications of CDAR thresholds [14].

Similarly, the mean cup-to-fovea over disc diameter (CF/DD) in our cohort was 2.48, with 6% of children exhibiting CF/DD > 3.0, questioning the appropriateness of this threshold for diagnosing small discs [2, 3].

Regarding interocular differences, only 0.5% of children in our study showed asymmetry >0.2, highlighting how rare pathological asymmetry is according to proposed thresholds of >0.25 [15]. Further exploration of asymmetry’s clinical significance in childhood is warranted to establish appropriate abnormal interocular difference levels.

In our study, a small head circumference below the 9^th^ percentile was associated with a small optic disc. It is unlikely that this is a proportional phenomenon given that CF/DD is a ratio, and large optic discs were not seen in those with large head circumference. One study reported smaller disc diameter in Australian children with small head circumference, which was associated with larger cup-to-disc ratio [16]. Small head circumference has been reported to be associated with neurodevelopmental abnormalities such as attention deficit, which, in turn, has been associated with subtle morphological changes in the optic nerve [17, 18].

Being underweight at age 11 was associated with large CDAR independent of maternal BMI, aligning with adult studies associating low BMI with smaller neuro-retinal rim area and larger vertical and area cup-to-disc ratios [19, 20]. Nutritional deficiencies may mediate this association, given the optic nerve’s vulnerability to deficiencies like B vitamins, folic acid, and proteins containing sulphur-containing amino acids [21]. In later life, glaucoma development has been linked with low vitamin A and vegetable fat intake [22].

Maternal smoking is a significant factor associated with small optic discs, likely due to its impact on foetal development and neuro-ophthalmic complications. Potential mechanisms include reduced placental blood flow through nicotine-induced vasoconstriction; foetal hypoxia from carbon monoxide binding to haemoglobin; vascular neogenesis; and endothelial function disturbance [23]. Additionally, direct toxic, ischaemic, or hypoxic effects on cell proliferation or migration during critical periods may contribute [23].

Studies of premature infants reported high mean CDAR and high prevalence of large cup-to-disc ratio, possibly influenced by factors precipitating preterm birth or immaturity-associated morbidity [24]. Immature apoptotic pruning of supernumerary fibres and extra-uterine environmental influences like oxygen delivery and carbon dioxide tension may cause excessive elimination of axons [24]. Persistence of large CDAR in older preterm children suggests limited catch up growth. Disentangling effects of gestational age from factors like low birth weight, retinopathy of prematurity, cerebral injury, and early life events is challenging. Cupped disc appearance in white matter injury may stem from axonal interruption via retrograde trans-synaptic degeneration, akin to optic nerve hypoplasia, with the differing appearance due to the timing of the insult [24, 25].

Some exposures like maternal age and parity, showed a lack of evidence of association.

### Implications and future work

This study contributes to a field primarily composed of small-scale investigations. We reveal that high cup-to-disc ratio and small optic discs may be more prevalent in children than previously believed. Therefore, it is crucial to scrutinize the clinical characteristics of children presenting with what is typically considered a high cup-to-disc ratio to ascertain whether these features are being overly referred, pathologized, or monitored.

The long-term prognosis of preterm high cup-to-disc ratio remains uncertain. While a larger ratio in adulthood is linked to a higher risk of glaucomatous optic neuropathy and associations have been reported between optic disc morphology and systemic neuro- and cardiovascular development in adults [26], the significance of high cup-to-disc ratio in preterm children as a form of optic nerve hypoplasia remains unclear. Further research is needed to elucidate the relationship between visual acuity and disc size.

Additionally, the association between small discs and head circumference raises questions about the need for further assessment in infants with smaller heads but not microcephaly. Work is needed to understand the relationship between timing and coordination of craniofacial growth and the morphology of optic fidelity.

Finally, addressing maternal smoking, a preventable risk factor, is essential to combat health disparities among socioeconomic groups.

## Conclusion

Retinal imaging of 3000 12-year-olds revealed a mean cup-to-disc area ratio of 0.21 and mean CF/DD of 2.48, consistent across sexes. Large CDAR affected 11%, linked to preterm birth and low child BMI. A small optic disc, found in 6% (one third bilateral), correlated with maternal smoking and small head circumference. These findings suggest the prevalence of high cup-to-disc ratio and small discs in children may be higher than previously thought. Optic nerve development may relate to extra-ocular growth and early-life factors, with distinct parameters in preterm children versus full-term counterparts.

## Summary

### What was known before

- It was unclear how common small discs and large cup-to-disc ratio were in the paediatric population and at what point such discs should be considered abnormal. Extreme phenotypes such as optic nerve hypoplasia had been associated with factors such as maternal age and parity, but these findings were necessarily based on small study numbers and it was unclear whether small discs per se would show similar associations.

### What this study adds

- We report that small discs and large cup-to-disc ratio may be more common in the paediatric population than previously thought (11% and 6% respectively). We suggest that the high prevalence of large cup-to-disc ratio and small discs in the general paediatric population may indicate that thresholds of normal for CDAR and disc size in children may benefit from re-consideration. We find that small postnatal head circumference and maternal smoking are associated with increased odds of a small optic disc. We find that being underweight and severe prematurity are associated with a CDAR > 3. Our work emphasises the importance of interventions to support maternal and infant nutrition and to reduce antenatal tobacco use.

## Supplementary information

Supplementary Table 1

## Data Availability

The data that support the findings of this study are not openly available due to reasons of sensitivity and are available by application to the ALSPAC (Explore data and samples | Avon Longitudinal Study of Parents and Children | University of Bristol) upon reasonable request. If you would like to discuss data access please email alspac-data@bristol.ac.uk (data).
