# Supplementary material for: Optic disc parameters and associations with early life exposures in over 3000 12-year-old children: findings from the ALSPAC cohort
Source: Eye (Lond). 2025 Feb 22;39(8):1592–8. doi: 10.1038/s41433-025-03716-2 (PMC12089515; doi:10.1038/s41433-025-03716-2)
Supplement: Supplementary file 1 — Supplementary Table 1 [file 41433_2025_3716_MOESM1_ESM.docx]

|  | **Gradable retinal photographs** | **Remaining ALSPAC cohort** | **p value*** |  |
| --- | --- | --- | --- | --- |
| **Demographic characteristics** |  |  |  |  |
| **Male** | 49.2% | 51.7% | 0.016 |  |
| n (total) | 1584 (3222) | 6056 (11720) |  |  |
| **Other ethnicity ^** | 4.0% | 5.4% | 0.002 |  |
| n (total) | 118 (2984) | 493 (9140) |  |  |
| **Maternal factors** |  |  |  |  |
| **A level education** | 44.1% | 32.5% | < 0.001 |  |
| n (total) | 1339 (3034) | 3061 (9431) |  |  |
| **Smoking T1 #** | 17.3% | 27.1% | < 0.001 |  |
| n (total) | 534 (3086) | 2745 (10127) |  |  |
| **Alcohol >2 units / day** | 0.7% | 0.5% | 0.52 |  |
| n (total) | 12 (1753) | 28 (5395) |  |  |
| **Age > 40 years** | 1.5% | 1.1% | < 0.001 |  |
| n (total) | 46 (3160) | 124 (10894) |  |  |
| **Birth factors** |  |  |  |  |
| **Gestation** |  |  | < 0.001 |  |
| **33-36 weeks' gestation** | 3.8% | 4.8% |  |  |
| n (total) | 119 (3160) | 516 (10783) |  |  |
| **<33 weeks' gestation** | 0.7% | 2.0% |  |  |
| n (total) | 23 (3160) | 221 (10783) |  |  |
| **Birth weight <2500g** | 4.2% | 6.3% | < 0.001 |  |
| n (total) | 130 (3121) | 681 (10762) |  |  |
| **Visual factors at 11 years** |  |  |  |  |
| **Acuity >0.3 LogMAR** |  |  | 0.55 |  |
| **Both eyes** | 0.2% | 0.3% |  |  |
| n (total) | 5 (2907) | 11 (3984) |  |  |
| **One eye** | 0.3% | 0.4% |  |  |
| n (total) | 9 (2907) | 16 (3984) |  |  |
|  |  |  |  |  |
| ** P value: chi-squared test for difference between those with retinal images and without.* | | | | |
| *^ Other ethnicity besides "white". Self-reported; incorporates the following categories as* | | | | |
| *determined by ALSPAC: Black Caribbean, Black African, Other black, Indian, Pakistani,* | | | | |
| *Bangladeshi, Chinese, Other.* |  |  |  |  |
| *# T1 is Trimester one.* |  |  |  |  |
